# Thermal Tracking in Mobile Robots for Leak Inspection Activities

**DOI:** 10.3390/s131013560

**Published:** 2013-10-09

**Authors:** Aitor Ibarguren, Jorge Molina, Loreto Susperregi, Iñaki Maurtua

**Affiliations:** IK4-Tekniker, Calle Iñaki Goenaga 5, Eibar 20600, Spain; E-Mails: jorge.molina@tekniker.es (J.M.); loreto.susperregi@tekniker.es (L.S.); inaki.maurtua@tekniker.es (I.M.)

**Keywords:** thermal image, leak detection, particle filter, tracking

## Abstract

Maintenance tasks are crucial for all kind of industries, especially in extensive industrial plants, like solar thermal power plants. The incorporation of robots is a key issue for automating inspection activities, as it will allow a constant and regular control over the whole plant. This paper presents an autonomous robotic system to perform pipeline inspection for early detection and prevention of leakages in thermal power plants, based on the work developed within the MAINBOT (http://www.mainbot.eu) European project. Based on the information provided by a thermographic camera, the system is able to detect leakages in the collectors and pipelines. Beside the leakage detection algorithms, the system includes a particle filter-based tracking algorithm to keep the target in the field of view of the camera and to avoid the irregularities of the terrain while the robot patrols the plant. The information provided by the particle filter is further used to command a robot arm, which handles the camera and ensures that the target is always within the image. The obtained results show the suitability of the proposed approach, adding a tracking algorithm to improve the performance of the leakage detection system.

## 1. Introduction

Efficient and effective maintenance is crucial for all kinds of industries. In the case of capital intensive investment industries, such as petrochemicals, the steel industry or power generation plants, it is even more relevant and has an important impact on the operation costs during the long lifecycle of their production means.

Automating inspection activities in industrial plants, especially in extensive plants, poses strong requirements from different points of view: a huge number of elements to inspect (pipes, valves, switches, pumps, vessels, motors, vibrating machinery, chillers, ovens, *etc.*), handling multiple sensors or special non-destructive testing equipment to be used (visual, ultrasonic, vibration, radiography, thermography, eddy current, noise analysis, gas sensors, *etc.*) and extensive production facilities that spread out for thousands of square meters, either in the vertical or horizontal, and risky working conditions for maintenance personnel, due to the presence of hazardous materials.

This paper presents part of the work performed in the MAINBOT European project. This project aims at developing service robot applications to autonomously execute inspection tasks in extensive industrial plants. The objective is to develop a surveillance robotic system able to detect the leakage of fluids using a vision system in the thermal and visible ranges. To validate the proposed solution, a solar plant of cylindrical-parabolic collectors is used, testing the approach in a very demanding environment from a mobile manipulation point of view.

The paper is organized as follows. Section 2 gives information about related works. Section 3 introduces the inspection task to be performed by the system. Section 4 presents the proposed approach, the architecture for leak inspection in solar thermal plants and autonomous navigation. Sections 5 and 6 are devoted to the leakage detection algorithm and the tracking system, respectively. In Section 7, the experimental results of the system are shown. Finally, Section 8 poses the obtained results, as well as the future work to be done.

## 2. Related Work

Robots have been used for maintenance tasks in a wide range of applications and environments. From preventive maintenance of high-voltage transmission power lines [1] to inspection of cables [2] and nuclear reactor pressure vessels [3] in the nuclear industry, autonomous systems are used in many industries in an attempt to improve maintenance tasks. Focusing on pipeline inspection, Suzuki *et al.* [4] propose an autonomous robot for industrial pipeline inspection by means of ultrasonic diagnosis equipment. In the same way, Camerini *et al.* [5] present an underwater inspection robot for offshore pipeline inspection, using the pipeline itself for guidance purposes. Even so, a few works add some tracking tools as particle filters to improve the inspection task.

Several works have also been performed using thermal images for tracking and detection. Jiping *et al.* [6] propose a target detection and tracking system based on different morphological operations. Senthil Kumar *et al.* [7] pose the fusion of thermal images with 2D images for tracking Unmanned Aerial Vehicles (UAVs), adding optical flow techniques, such as LucasKanade and HornSchunck methods. Finally, Padole and Alexandre [8] propose the use of particle filters for human tracking with thermal images, using motion information to feed the particle filter. The presented work also proposes the use of particle filtering, although it uses the whole image information and dynamics to feed the particle filter, not only the motion information.

## 3. Task Specification

**Figure 1.**
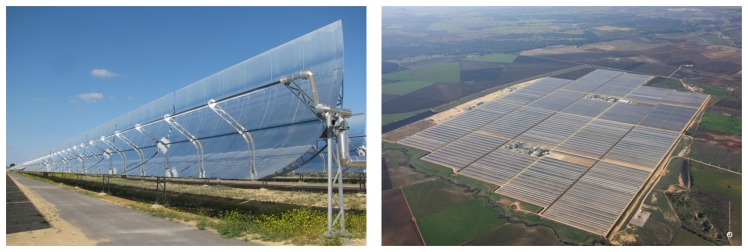
Valle 1 and 2 solar thermal power plants.

Valle 1 and 2 are two solar thermal power plants, whose promoter and owner is Torresol Energy Investments, S.A., and which are located in San José del Valle (Spain); see Figure 1. Valle 1 and 2 are two adjacent solar thermal power plants that generate electricity by means of cylindrical-parabolic collectors.

The solar field is composed of nearly 7,500 parabolic cylinder collectors. These collectors transport a Heat Transfer Fluid (HTF), which absorbs the solar energy. HTF circulates at a high temperature (around 390 °C) inside the absorber tubes, which is used after to heat the molten salts to generate steam in the Steam Generation System (SGS).

Swivel joints are critical points where leakages may happen (this is the point where the collector tube connects with the infrastructure of pipes that are deployed all over the plant). HTF leakages are not desirable, as oil losses may be unsafe, due to the high temperatures reached in the solar power plant. Early detection and prevention of leakages is a key issue for the maintenance of those kinds of facilities. Even so, the huge area of solar power plants makes it difficult to perform proper maintenance, due to the large amount of kilometers of collectors and pipelines, as well as the hazardous environment with the really high temperatures reached in its elements.

Nowadays, the inspection is performed by human operators using a thermographic camera while they travel along different parts of the solar plant by car through poorly asphalted and dirt roads. The inspection is carried out while the vehicle is moving, looking at the screen to analyze the thermal image and detecting leakages. Even so, the operator must inspect the image for long time periods (around 2 h) while correcting the the pose of the camera to keep the pipeline in the field of view when irregularities in the terrain appear, which may lead to leakages being left undetected.

## 4. Proposed Approach

Based on the task specifications posed in the previous section, this paper proposes an autonomous robotic system to perform the pipeline inspection for early detection and prevention of leakages. The autonomous robot patrols along the power plant while inspecting the collectors using thermographic images to identify the leakages. The inspection system continuously analyzes the images in order to monitor the status of the elements, the tube in this case, and highlight anomalies.

Initially, a path is defined for the robotic platform based on a hybrid map approach mixing topological graphs with local occupancy grids. Using the information provided by a GPS/IMU sensor, the robot executes the planned path. During this execution, the system takes advantage of a local metric planner to avoid obstacles and follows the initial path as close as possible.

Even so, irregularities in the terrain (poorly asphalted road and dirt road) make it difficult to perform the inspection using a fixed camera mounted on the robot, as the pipeline can be out of range when slopes and bumps are found on the road. Taking this into account, the addition of a robotic arm is proposed to allow the manipulation of the thermal camera and to track the pipeline through the inspection task. The aim is to establish a coordination between the thermographic inspection and the robot arm movements in order to keep the objective in the field of view of the camera.

The next sections will give information about the used hardware and system architecture, including the different software units defined within the system and the navigation system.

### 4.1. Architecture

**Figure 2.**
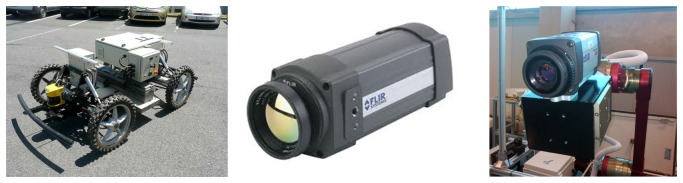
Robotic platform and thermographic camera.

The architecture presented in this paper is based on the specifications and work performed in the MAINBOT project. The main robotic platform used is a RobucarTT developed by Robosoft (http://www.robosoft.com/), designed for outdoor environments and with the Ackermann steering geometry. The robot includes a GPS/IMU sensor for localization purposes, which provides the robot position with an accuracy of 0.20 m. The platform also has a robotic arm attached to it, which is employed in this case to handle a thermographic camera, specifically, an FLIRThermoVision® A20. This thermographic camera is used for leakage detection, as well as for tracking purposes. Figure 2 shows the physical elements of the architecture.

**Figure 3.**
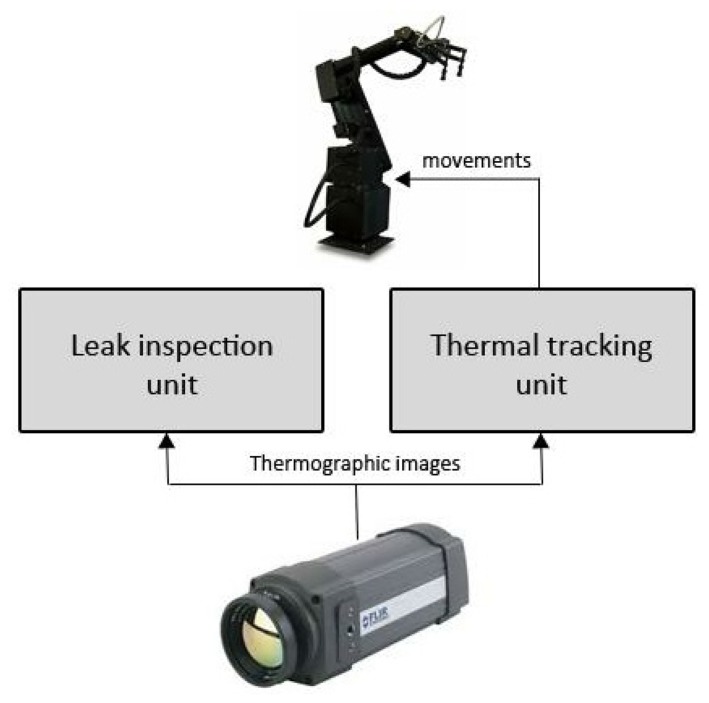
Software architecture: leak inspection unit and thermal tracking unit.

There are two main tasks to be performed by the robotic platform while the predefined path is being executed by the robotic platform: on the one hand, to inspect the pipelines to search for leakages; on the other hand, to track the pipeline in order to send movements to the robotic arm and to maintain the pipe in the field of view of the camera. To this end, as shown in Figure 3, two different modules have been defined in the software architecture:

- **Leak inspection unit**: the unit in charge of receiving images from the thermographic camera and analyzing them in order to detect leakages in the collectors. Based on different morphological operations in the image, the unit is able to detect the leakages in the collectors in an accurate way.
- **Thermal tracking unit**: the unit in charge of tracking the pipeline and commanding the robotic arm to keep the pipe in the field of view of the camera. A particle filter-based tracking system is proposed, because to its capacity to accurately model the underlying dynamics and its rapid adaptation to changing signal features.

Based on this architecture, the system is able to (a) inspect the pipelines and detect leakages and (b) maintain stable the detection process by means of the tracking process, overcoming the problems derived from the irregularities of the terrain, while the mobile robot executes the planned path for inspection.

### 4.2. Autonomous Navigation

The autonomous navigation approach of the MAINBOT project is based on the use of a hybrid map consisting of a topological graph overlaid with local occupancy grids. Since the workspace is a large area, the overall plan is formed on a topological graph, as planning in a large metric map quickly becomes unwieldy. However, local metric information is used for achieving precise localization (needed for some operations) and obstacle avoidance. Hence, the path planning is performed in two steps:

- The overall plan is created in the topological graph, using Dijkstra's algorithm. This is the basis for the low level planning.
- The robot navigates locally using local metric maps and a search-based planning algorithm.
  – The global metric planner integrated in MAINBOT generates a path from the current position to a desired goal by combining a series of short, kinematically feasible “motion primitives”. Planning is done in x, y and theta dimensions, resulting in smooth paths that take robot orientation into account. This is especially important for a RobucarTT robot, as it has nonholonomic constraints (*i.e.*, due to the Ackermann configuration).
  – The local metric planner can be seen as a controller that drives a mobile base in the plane.
  – An execution component called “*move base*” links the global and local planners to achieve the metric navigation.

This navigation approach allows for creating paths for pipeline inspection in two steps, using the “*move base*” component to execute the defined path as accurately as possible. The autonomous navigation module has been developed using ROS (http://www.ros.org) (Robot Operating System) libraries and packages.

## 5. Leak Inspection Unit

The aim of this unit is the detection of leakages in the collectors based on information provided by a thermographic camera. To this end, initially, (a) the image is analyzed, searching for a pipeline section. Once the pipeline has been found; (b) the section is inspected to detect abrupt changes in the temperature, which indicate that there are leakages in the collectors.

In this process, as the first step, the object parameters of the thermal camera must be fixed. To extract the temperature information from an image, the output data of the thermal camera must be interpreted based on the correct fixing of parameters, such as emissivity, object distance or reflected temperature.

The emissivity is a surface property that states the ability to emit energy; it is expressed as the ratio of the radiation emitted by a surface to the radiation emitted by a blackbody. Emissivity is a unitless quantity and spans from zero to one.

Following the energy conservation law, all energy exchange is compensated mutually: the flux incident, Φ*_i_*, is equal to the flux reflected, $\Phi_{r}^{\prime }$, absorbed, Φ*_a_*, and transmitted, Φ*_t_*:

(1) $$\Phi_{i}=\Phi_{r}^{'}+\Phi_{a}+\Phi_{t}$$

In general cases, terms on the right side of Equation (1) are specifically weighted following particular radiative properties related to reflection (*ρ*), absorption (*α*) and transmission (*τ*).

These properties are linked together considering the flux exchanges on a semitransparent object in its environment for which:

(2) ρ+α+τ=1

The general form of Kirchhoffs law provides a link between the absorption and emission processes and, thus, between emissivity and absorbance, since:

(3) $$\varepsilon (\lambda ,\theta^{\prime },\phi^{\prime })=\alpha (\lambda ,\theta ,\phi )$$

The object distance is defined as the distance from the camera to the surface. Finally, reflected temperature indicates the temperature reflected by the surface of an object.

In the case of the glass that covers the collector, it has a high transmissivity, around 94% (0.94), so the emissivity is fixed to 0.04. The estimated reflected temperature is fixed empirically to 10 °C and the object distance to 10 m, based on the features of the environment and the solar plant.

**Figure 4.**
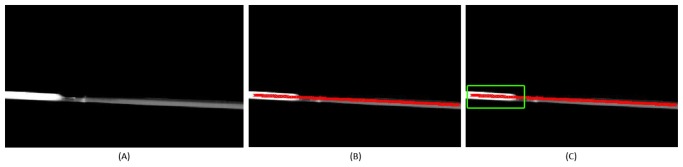
(**A**) Thermographic image; (**B**) Pipeline detection; and (**C**) Leak detection.

Once the settings are established, the camera processes the thermal information and provides an image where pixels give information about the temperature. These values of temperature are normalized to gray values performing a thermal adjustment between the minimum and maximum values defined (in our case, between 100 °C and 300 °C), as shown in Figure 4A.

This image is then thresholded, highlighting the pixels with temperatures above 100 °C, as they are related with the pipelines. Skeletonization/Medial Axis Transform [9] is applied to this image, detecting the longest straight section in the image, as illustrated in Figure 4B. This straight section is then analyzed to detect temperature changes along the pipeline.

**Figure 5.**
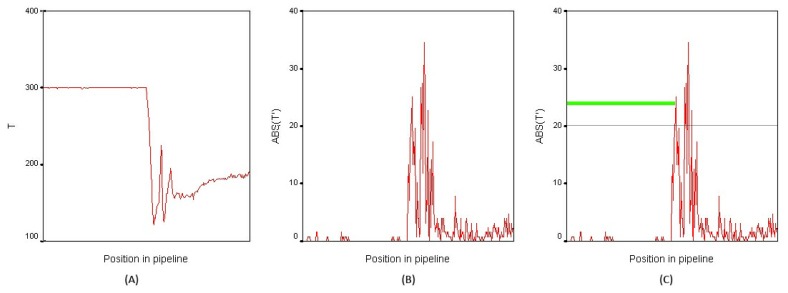
(**A**) Temperature along the pipeline; (**B**) Absolute value of the first derivative; and (**C**) Detection on the plot.

As the first step to detect the temperature changes along the collector, the temperatures of the previously obtained section are stored, as shown in Figure 5A, where the temperature along the collector is plotted. Based on these data, the absolute value of the first derivative is calculated as:

(4) $$\text{ASB}(T^{\prime })=|T_{i+1}-T_{i}|,i=[1..N-1]$$

where *T_i_* is the temperature of pixel *i* of the pipeline section.

The information about the absolute value of the derivative is used to divide the section into different parts, which have similar temperature, defining a Δ*T* threshold (minimum change) to perform this division. An important consideration is the fact that the joints of different sections of the collector are metallic and have a high reflectivity, so the obtained temperature is unstable at this point and must be filtered. In order to filter them, an approximate resolution of the image is estimated and short parts with abrupt change of temperature are removed, defining a minimum width of the pipeline part. This allows us to filter short pipeline parts with abrupt temperature changes (joints), detecting real leakages, which fill a wider space on the image. Once the pipeline is divided, the mean temperature of each subsection is computed. The pipeline parts above the maximum temperature are labeled as leakages, as highlighted in green in Figures 4C and 5C.

Based on this algorithm, the *Leak Detection Unit* is able to raise alerts when leakages are detected while the robot is patrolling along the collectors.

## 6. Thermal Tracking Unit

Irregularities in the terrain can make it difficult to perform a correct leak inspection, as pipelines can be out of the field of view of the camera when slopes and bumps are found along the road. To overcome this problem, a tracking system is proposed based on particle filtering. This tracking system follows the target pipeline through thermographic image sequences, and it is able to generate movement commands when the target is reaching the edge of the image. The next lines will give information about all the elements of the particle filter for thermal tracking.

### 6.1. Particle Filter

Particle filters [10,11], also known as Sequential Monte Carlo methods (SMC), are sequential estimation techniques that allow estimating unknown states, *x_t_*, from a collection of observations *z*_1:_*_t_* = {*z*_1_, …, *z_t_*}. The state-space model is usually described by state transition and measurement equations:

(5) $$x_{t}=f_{t}(x_{t-1},v_{t-1})$$

(6) $$z_{t}=g_{t}(x_{t},u_{t})$$

where *f* and *g* are the state evolution and observation model functions, respectively, and *v_t_* and *u_t_* denote the process and observation noise, respectively.

Based on the previous equations, particle filters allow for approximating the posterior density (PDF) by means of a set of particles, ${\left\{x_{t}^{\left(i\right)}\right\}}_{i=1,\ldots ,n}$, using equation:

(7) $$p(x_{t}|z_{1:t})=\sum_{i=1}^{N}\omega_{t}^{\left(i\right)}\delta (x_{t}-x_{t}^{\left(i\right)})$$

where each particle, $x_{t}^{\left(i\right)}$, has an importance weight, $\omega_{t}^{\left(i\right)}$, associated with it and *δ* is the Kronecker delta. These weights are computed following equation:

(8) $$\omega_{t}^{\left(i\right)}=\omega_{t-1}^{\left(i\right)}\frac{p(z_{t}|x_{t}^{\left(i\right)})p(x_{t}^{\left(i\right)}|x_{t-1}^{\left(i\right)})}{q(x_{t}^{\left(i\right)}|x_{0:t-1}^{\left(i\right)},z_{0:t})}$$

where $p(z_{t}|x_{t}^{\left(i\right)})$ is the likelihood function of the measurements, *z_t_*, and, finally, $q(x_{t}^{\left(i\right)}|x_{0:t-1}^{\left(i\right)},z_{0:t})$ is the proposal density function.

Based on the previously presented equations, the particle set evolves along time, changing the weights of the particles and resampling them in terms of the observations.

Particle filtering provides a robust tracking framework when dealing with non-linear and non-Gaussian state and observation functions, as it considers multiple state hypotheses simultaneously.

### 6.2. Particle Filtering for Thermal Tracking

In the first step, a tracking process has been defined in an attempt to maintain the object to be analyzed in the field of view of the thermal camera. A particle filter-based tracking is proposed, allowing us to correct the position of the robotic arm to maintain the pipe in the center of the image for a further analysis. In an attempt to develop a thermal tracking system for multiple detection tasks, the particle filter has been generalized to allow the tracking of objects with different shapes and configurable for each tracking process (easy reconfiguration for similar scenarios), although the system modeling explained in the next lines is tuned for the presented environment and posed problem.

#### 6.2.1. System Modeling

In the presented scenario, the tracking process is able to follow a pipeline in successive frames. The state of the process is defined as:

(9) $$X_{t}={\left[x_{t},y_{t},l_{t},\alpha_{t}\right]}^{T}$$

where *x_t_* and *y_t_* are the *x* and *y* pixel coordinates of the center of the pipeline in the image, *l_t_* is the length of the pipeline and *α_t_* is the orientation of the pipeline in time *t*.

Additionally, the state transition is defined as:

(10) $$X_{t+1}=X_{t}+\Delta_{t}{\dot{X}}_{t}+V_{t}$$

(11) $${\dot{X}}_{t}={\left[{\dot{x}}_{t},{\dot{y}}_{t},{\dot{l}}_{t},{\dot{\alpha }}_{t}\right]}^{T}$$

where Δ*_t_* is the time step,*Ẋ_t_*is the dynamic part describing the variation of the state elements and *V_t_* is an additive, zero mean Gaussian noise.

#### 6.2.2. Likelihood Evaluation

For the likelihood evaluation, initially, the thermal image is analyzed to highlight the parts with a predefined temperature range, in this case, the temperature of the pipe to be tracked. To this end, thresholding and Skeletonization/Medial Axis Transform algorithms have been used, as in the *leak inspection unit*. From this step, a set of *N* connected regions are extracted; *N* possible pipe sections. Those regions form the observation, *Z_t_*, where each region, $z_{i}^{t}$, is defined by their center in pixel coordinates, length and angle:

(12) $$Z_{t}=z_{i=1¨N}^{t}$$

(13) $$z_{i}^{t}={\left[x_{i}^{t},y_{i}^{t}l_{i}^{t},\alpha_{i}^{t}\right]}^{T}$$

For the likelihood evaluation, initially, the distance between the particle and each of the observed regions is calculated using the pixel coordinates of the center, length and angle as:

(14) $${\text{dist}}_{i}=\lambda \sqrt{{\left(x_{t}-x_{i}^{t}\right)}^{2}+{\left(y_{t}-y_{i}^{t}\right)}^{2}}+\beta |l_{t}-l_{i}^{t}{|}^{2}+\gamma |\alpha_{t}-\alpha_{i}^{t}{|}^{2}$$

where λ, *β* and *γ* are coefficients to weigh the importance of the pixel coordinates, length and angle, respectively. The distance between the particle and the observation is then calculated as the minimum distance between the particle and the *N* regions found:

(15) $$\text{dist}(X_{t},Z_{t})=\min({\text{dist}}_{i}),i=[1‥N]$$

Finally, the likelihood is calculated as the exponential of the distance, as shown in the next equation:

(16) $$P(Z_{t}|X_{t})=e^{-\text{dist}(X_{t},Z_{t})}$$

Based on the presented likelihood evaluation, the particle filter estimates iteratively the process state as presented in the next paragraphs.

#### 6.2.3. Particle Filtering Procedure

To initialize the process, when the first pipe is detected, a set of N random particles is drawn around its position and with its scale and orientation. Afterwards, the procedure of the particle filter is given as:

- Find the object in the initial thermal image and initialize N particles, $X_{0}^{\left(i\right)}$, with random samples around it, where $\omega_{0}^{\left(i\right)}=1/N$;
- If *ESS* < *threshold* (*effective sample size*), draw N samples with *selection with replacement*;
- Predict $x_{t}^{\left(i\right)}=x_{t-1}^{\left(i\right)}+\upsilon_{t-1}$;
- Update importance weights $\omega_{t}^{\left(i\right)}=\omega_{t-1}^{\left(i\right)}P(Z_{t}|X_{t})$; 5: Normalize weights $\omega_{t}^{\left(i^{'}\right)}=\omega_{t}^{\left(i\right)}/\sum_{j=1}^{N}\omega_{j}^{\left(j\right)}$;
- Set *t* = *t* + 1, go to Step 2.

In this procedure, ESS [12] (effective sample size) is calculated as:

(17) $$c\upsilon_{t}^{2}=\frac{\upsilon ar(\omega_{t}^{\left(i\right)})}{E^{2}(\omega_{t}^{\left(i\right)})}=\frac{1}{N}\sum_{i=1}^{N}{\left(N\omega_{t}^{\left(i\right)}-1\right)}^{2}$$

(18) $${\text{ESS}}_{t}=\frac{N}{1+c\upsilon_{t}^{2}}$$

where *N* is the number of particles and $\omega_{t}^{\left(i\right)}$ is the weight of particle *i* in time *t*.

Based on this discrete approximation of the posterior probability, the object is tracked along the inspection process.

## 7. Experimental Results

**Figure 6.**
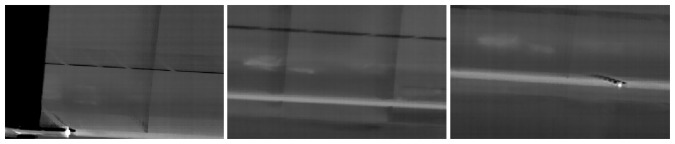
Sequence of thermographic images on Valle facilities.

To test the suitability of the proposed approach, a set of experiments have been designed trying to asses both the leakage detection algorithm and the tracking system. To this end, a database of image sequences was created in Valle facilities (see Figure 6) using actual production means and replicating the behavior of the maintenance robot:

- Recording of collectors and pipelines using a thermal camera 10 km at night, as performed now by human operators, divided in sequences of 150 m (half loop of collectors).
- Camera placed on a vehicle circulating at a speed of 20 km/h through a terrain with irregularities.
- Real leakages appearing in the images.

Based on this real data, two different experiments have been performed. The next lines give information about each experiment and the obtained results.

### 7.1. Results of the Leak Detection

An efficiency of the leak detection has been tested using 60 different thermal sequences. In those 60 sequences, human operators found a total of seven leakages during the recording session, which were labeled in the database. Those 60 sequences were analyzed by the previously presented algorithm, searching for leakages. A threshold, Δ*T*, of 70 °C was established for the detection algorithm empirically based on the gathered data. In order to have numeric data, a mean value for each stretch of the tube with temperature change (filtering joints) was also saved.

**Figure 7.**
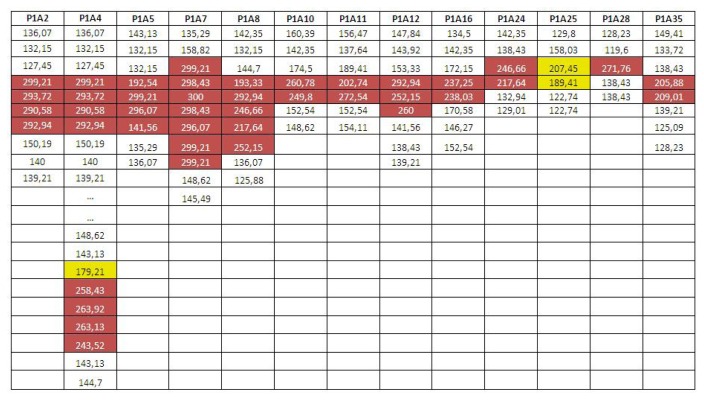
Example of changes in temperature in recorded image sequences.

Figure 7 shows an example of the output of the algorithm while performing the validation test, where each column is related with a sequence. The first row indicates the image sequence ID, while the next rows show the mean temperature of the pipe section along the collectors where abrupt changes of temperature can be observed. Pipe parts with a high temperature (leakages) are marked in red.

The algorithm found all the leakages labeled by human operators, obtaining 100% sensitivity. Besides, six more leakages were found along the sequences, leakages that match exactly with the previous leakage patterns. Based on the similarity and after an analysis of the new leakages, they could be considered as real leaks that were missed by the human operators during visual inspection.

### 7.2. Results of the Tracking Process

**Figure 8.**
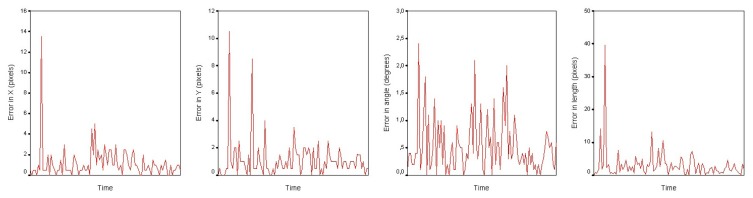
Tracking error during a recorded sequence in position in X and Y orientation and length.

To test the efficiency of the particle filter for tracking, 10 different image sequences have been used, each of them with 200 images approximately (a total of 2,000 images). For each sequence, the particle filter has been initialized using the first pipeline appearance, and the pipeline has been tracked through the rest of the images. In this experiment, the manipulation of the robotic arm has been left out of the scope of this manuscript, as the sequences have been recorded by a human operator from a car and it is not possible to simulate the arm movements. For each frame, the error between the output of the particle filter and the labeled images has been computed, as shown in Figure 8. Images show peaks in the error plots, derived from the image noise and irregularities in the terrain.

Specifically, the experiment measures the tracking error in position, angle and length of the pipeline, using different parameters for the particle filter. Those are the specifications of the experiment:

- Six different particle filter configurations have been set up, mixing different state estimation methods and numbers of particles. Specifically, the state estimation methods are:
  – Best particle (the one with maximum weight);
  – Robust mean with the 10 particles with the maximum weight;
  – Weighted mean using the whole particle set;
- For each configuration, the previously cited image sequences have been used, tracking the pipeline through around 2,000 images; the mean error and standard deviation of the position, angle and pipe length has been measured for all the configurations;
- In the likelihood evaluation step, the same values for coefficients λ, *β* and *γ* have been used, applying values that give a similar weight to position, angle and length.

Table 1 shows the obtained results. The first column describes the number of particles and the estimation method, the second and third columns, the mean (*μ*) and the standard deviation (*σ*) of the error in the position and the fourth and fifth columns, the mean (*μ*) and the standard deviation (*σ*) of the error in the angle, and finally, the last columns show the mean (*μ*) and the standard deviation (*σ*) of the error in the length.

Results show a better performance of the particle filter with a set of 1,000 particles, improving the mean error and standard deviation in almost all the configurations. The addition of a bigger set of particles could decrease the error rates, although it would be necessary to optimize the computational time as much as possible to ensure a suitable frame rate. In the case of the state estimation methods, the best particle and robust mean methods show very similar results, overcoming in any case the errors of the weighted mean.

## 8. Conclusions and Future Work

This paper presents an autonomous leakage detection system for maintenance tasks in extensive industrial plants, including a leakage detection system. Based on the information provided by a thermographic camera, the system is able to detect leakages in pipelines and collectors of thermal power plants. This system is enhanced by a particle filter-based tracking system, used to maintain the target in the field of view of the camera and stabilize the detection process when irregularities are found along the road.

The results show a high success rate of the leakage detection unit, reaching 100% sensitivity (on data labeled by operators) and detecting even more leakages on the recorded sequences. The use of thermographic information allows for detecting the fluids leaked from the collectors, taking advantage of morphological operations to highlight the leaks in the thermal images.

Besides, a tracking system has been added to manage the thermographic camera and to avoid the loss of the target. The particle filtering for tracking has shown a position error of less than 1.5 pixels and less than a 0.5° error in the angle. It also allows for modeling the system in a simple and effective way, adapting rapidly to the changing image features and data noise.

As further work, there are two main paths to follow: on the one hand, to add the robot arm movements and to mix the information extracted by the leak inspection unit and thermal tracking unit with the navigation module to create a complete system that takes into account the navigation information in the inspection task and *vice versa*. In the same way, it would also be interesting to test the tracking system in similar inspection tasks where different elements of the solar plant are analyzed (valves, vessels, *etc.*) and observe its suitability.

## Figures and Tables

**Table 1.** Results of the Tracking Process.

	**Pos. Error (pix)**		**Angle Error** (°)		**Length Error (pix)**	
		
	*μ*	***σ***	*μ*	***σ***	*μ*	***σ***
500 - Best particle	1.80	1.27	0.96	0.86	3.16	2.58
1,000 - Best particle	**1.42**	**0.95**	0.72	0.63	2.94	**2.51**
500 - Robust mean 10	1.82	2.11	0.51	0.41	2.86	5.35
1,000 - Robust mean 10	1.57	1.83	**0.41**	**0.34**	**2.65**	5.66
500 - Weighted mean	6.34	5.39	0.43	0.36	3.67	7.68
1,000 - Weighted mean	6.11	4.67	0.35	0.38	3.45	6.64
